# Genetic Distinctness and Diversity of American Aberdeen Cattle Compared to Common Beef Breeds in the United States

**DOI:** 10.3390/genes14101842

**Published:** 2023-09-22

**Authors:** Nayan Bhowmik, Travis Seaborn, Kris A. Ringwall, Carl R. Dahlen, Kendall C. Swanson, Lauren L. Hulsman Hanna

**Affiliations:** 1 Department of Animal Sciences, North Dakota State University, Fargo, ND 58108, USA; 2 School of Natural Resource Sciences, North Dakota State University, Fargo, ND 58108, USA; 3 Dickinson Research Extension Center, North Dakota State University, Dickinson, ND 58601, USA

**Keywords:** lowline cattle, genetic diversity, population structure

## Abstract

American Aberdeen (AD) cattle in the USA descend from an Aberdeen Angus herd originally brought to the Trangie Agricultural Research Centre, New South Wales, AUS. Although put under specific selection pressure for yearling growth rate, AD remain genomically uncharacterized. The objective was to characterize the genetic diversity and structure of purebred and crossbred AD cattle relative to seven common USA beef breeds using available whole-genome SNP data. A total of 1140 animals consisting of 404 purebred (*n* = 8 types) and 736 admixed individuals (*n* = 10 types) was used. Genetic diversity metrics, an analysis of molecular variance, and a discriminant analysis of principal components were employed. When linkage disequilibrium was not accounted for, markers influenced basic diversity parameter estimates, especially for AD cattle. Even so, intrapopulation and interpopulation estimates separate AD cattle from other purebred types (e.g., Latter’s pairwise F_ST_ ranged from 0.1129 to 0.2209), where AD cattle were less heterozygous and had lower allelic richness than other purebred types. The admixed AD-influenced cattle were intermediate to other admixed types for similar parameters. The diversity metrics separation and differences support strong artificial selection pressures during and after AD breed development, shaping the evolution of the breed and making them genomically distinct from similar breeds.

## 1. Introduction

Once the cattle genome sequence was established [1], the ability to understand the genetic structure of different cattle breeds and their crosses could be inferred more easily [2]. The usefulness of single nucleotide polymorphism (SNP) markers in genetic structure studies has also been documented [3,4]. The application of these concepts more recently has focused primarily on European cattle breeds and the genetic divergence of these breeds from *Bos indicus* breeds [5,6]. Researchers continue to genetically characterize populations in an effort to document available genetic diversity and resources for conservation as well as food security within and across countries [7,8,9,10]. Particular interest has also focused on understanding the genetic structure and diversity of indigenous cattle breeds, often compared to the *B. taurus* and *B. indicus* breeds used in commercial beef production [6,7,9,10,11,12,13,14].

American Aberdeen (AD) cattle in the USA, formerly referred to as American Lowline, descend from an Aberdeen Angus herd originally brought to the Trangie Agricultural Research Centre (TARC), New South Wales, AUS, in 1929. In 1963, TARC started using the herd for selection research and closed the herd to outside genetics after final bull purchases in 1964 [15]. From 1963 to 1973, the herd was selected based on adjusted yearling weight and conformation. In 1974, TARC created three distinct herds from available cattle based on yearling growth rate (low, control, high) and selected replacements using this trait [15]. Results showed that after 15 years of selection on yearling growth rate, the low and high lines differed by 28% in weaning weight, yearling weight, and yearling gain [15,16]. Subsequent studies showed correlated responses in body size measures, rate of maturation, and body composition [16,17,18], providing evidence that these traits could be selected and indicating that the lines created were genetically different. Even so, information is lacking on the genomic differences or similarities to other cattle breeds.

A group of seven producers formed the Australian Lowline Cattle Association (ALCA) in 1992 using cattle purchased from dispersal sales of the low line cattle at TARC [19]. In 1996, the first ALCA registered heifer was imported into North Dakota (ND), USA [20]. Since then, the AD breed has expanded into almost 900 operations across 48 states and internationally [21]. The use of AD in the USA, particularly in crossbreeding systems, is increasing. Therefore, understanding the population structure and genetic diversity of the AD breed is necessary in order to meet production needs in various environments, allow effective management and sustained genetic improvement, and facilitate rapid adaptation to changing environments and breeding objectives [8,22].

A unique research population of purebred and crossbred cattle at the North Dakota State University (NDSU) Dickinson Research Extension Center (DREC) provides an opportunity to explore the genetic structure and diversity of the AD breed relative to other common beef breeds used in the same herd. This herd includes AD cattle with pedigree tracing to TARC via animals imported into the USA from ALCA. The closest connection of DREC AD cattle was two matings removed from ALCA registered animals and three matings removed from TARC animals. The DREC herd also includes purebred and crossbred cattle from seven other USA breeds. The objective of this study was to characterize the genetic diversity and structure of purebred and crossbred AD cattle relative to seven common beef breeds in the USA using available whole-genome SNP data. Given the selection pressure placed on AD cattle in their development as well as an established breed registry for more than 10 years, we hypothesized that AD cattle would clearly differentiate themselves from other breeds, even from their breed of origin, black Angus (AN).

## 2. Materials and Methods

### 2.1. Animals

A long-term project commenced in 2014 based on the current (base) herd at the NDSU DREC ranch located near Manning, ND. This project included organizing available tissue samples (ear punches) from past animals and collecting blood samples on current animals for DNA extraction. The admixed base cow herd comprised two distinct groups due to management and selection decisions at the NDSU DREC. The first group, called the “beef herd”, consisted of moderate- to large-framed black AN, Red Angus (AR), Gelbvieh (GV), Shorthorn (SH), or Simmental (SM)-influenced cows produced from using registered purebred bulls of those respective breeds. The second group, called the “range herd”, consisted of small- to moderate-framed AD-influenced cows, which were produced from crossing AD bulls with AN, AR, GV, SH, or SM-influenced heifers (typically produced from the beef herd). Admixed cows (*n* = 202) from these two herds (i.e., the base herd) along with herd-raised bulls (*n* = 2) and steers (*n* = 48) with available samples were included in this study (*n* = 252; ADMX-I). The NDSU DREC had records of dams and suspected or known sire for each calf born. Known or suspected breeds were also available for all admixed bulls and dams purchased since the early 2000s. Therefore, expected breed fractions based on recorded mating were identified for all ADMX-I animals with genotypes. Based on known pedigrees, many of these ADMX-I animals (*n* = 168, 66.7%) also had some level of Hereford (HH, regardless if horned or polled) influence in their ancestry, even though HH bulls were not being used at the time the project commenced and, thus, DNA samples from HH bulls were not available. The HH ancestry influenced daughters and granddaughters retained in the long-term study (see below). Furthermore, based on known pedigree, a small portion of ADMX-I females had up to 50% unknown ancestry (*n* = 29, 11.5%) due to incomplete records. Because of this, other *B. taurus* breeds not described could be influencing the population because they were not recorded in breeding and pedigree records, although the most probable breeds are those previously described, particularly AN. Due to the climate of ND, it was known that *B. indicus* breeds or composite breeds with *B. indicus* influence were not present in the population.

Daughters produced in the base herd from 2014 to 2017 (*n* = 273; ADMX-II) had samples collected for DNA extraction as part of a long-term study focused on longevity traits. These daughters were bred with either AR or AD bulls. A subset of granddaughters produced from 2016 to 2019 were also retained as part of the long-term project (*n* = 213; ADMX-II) and had samples collected for DNA extraction. The ADMX-II group did consist of 4 females (3 daughters and 1 granddaughter) with recorded Limousin (LM) sires, which was not seen in ADMX-I. No purebred LM samples were available, and collection was not possible since the bulls had been sold prior to the project commencing. 

In addition to these admixed populations, DNA samples were available from 107 animals (bulls and cows used in the NDSU DREC herd) that were known purebreds and registered within 6 breeds (AD, AN, AR, GV, SH, and SM). In 2019 and 2020, a set of registered purebred HH bulls (*n* = 11) were purchased as part of the second phase in the long-term project and were included in this study to serve as a reference for the HH breed seen in admixed animals. Additional purebred samples were identified using the Web-Interfaced next generation database dedicated to genetic Diversity Exploration (WIDDE) [23] to offset some breeds having a low sample size, as well to as reduce any bias present due to high relatedness in the NDSU DREC population. Purebred animals in WIDDE for AN, AR, GV, HH, LM, SH, and SM breeds were selected. The WIDDE algorithm automatically reduces markers to match across genomic marker panels using marker name and UMD 3.1 map coordinates [1]. The only quality filter used before exportation on WIDDE purebreds was to ensure each individual had at least a 95% marker call rate for available markers. In total, an additional 286 individuals were included and 46,387 markers were exported for each WIDDE individual in PLINK *.ped* and *.map* file formats [24] with SNP ID setting as Illumina names. WIDDE cattle exported were previously included in studies by Matukumalli et al. [25], Illumina, Inc. [26], or both.

### 2.2. NDSU DREC Population DNA, Genotyping, and Parentage Testing

Extraction of DNA was performed from blood samples collected via jugular venipuncture (*n* = 844) or ear tissue samples (*n* = 21) using the Qiagen DNeasy extraction kit protocol (QIAGEN, Hilden, DEU, Germany). The quality of DNA was checked using a Synergy H1 microplate reader (BioTek, Winooski, VT, USA), then stored at −80 °C until plating for shipment. All DNA samples were thawed, resuspended, plated to achieve 500 ng of DNA, then dried at 80 °C entirely in 96-well plates before shipment. Once samples arrived at Neogen GeneSeek laboratory, technicians resuspended samples for genotyping based on the concentration needed for their genotyping procedures. Animals were genotyped using the GeneSeek Genomic Profiler 150K (GGP150K; *n* = 727) or 100K (GGP100K; *n* = 138) for Beef Cattle (Neogen GeneSeek, Inc., Lincoln, NE, USA). Total SNP markers genotyped on the GGP150K panel were 139,376, which were mapped using UCD 1.2 assembly [27]. Only SNP markers mapped to autosomes were used in analyses (*n* = 133,155), which excluded 342 pseudoautosomal region, 5283 X chromosome, 62 Y chromosome, and 534 unmapped markers. The animals genotyped on the GGP100K (due to changes at Neogen GeneSeek, Inc.; *n* = 95,256 total SNP markers) were aligned to the GGP150K panel to identify matches. A total of 67,281 SNP markers were identified on both panels without duplication. Another 1153 SNP markers were identified as duplications and present on one or both panels given UCD 1.2 assembly coordinates. Duplicates were evaluated based on call rate (CR), minor allele frequency (MAF), and allele alignment within the NDSU DREC population, then merged so that a single marker represented that assembly coordinate with the best CR (95% or higher) and MAF (5% or higher), given available genotypes (*n* = 62 unique markers retained; 67,343 total markers). 

Parentage testing based on recorded mating for genotyped animals was used to identify whether incorrect parent assignments were common and if those incorrect parent assignments changed expected pedigree-based breed fractions. Not all genotyped individuals had genotyped parents, therefore this effort was taken to ensure that assuming breed population strata from mating records was a reasonable strategy. Parentage for genotyped animals was confirmed or updated once genotypes became available. Genotyping occurred from 2015 to 2021 in batches based on sample availability. Parentage analysis of animals with genotypes available was conducted in stages using possible genotyped parents for a given set of animals and markers filtered using global quality control standards of MAF > 5%, CR > 95%, and an exact Hardy–Weinberg equilibrium (*p* > 0.0001) test following Wigginton et al. [28] in current R software [29] within RStudio [30] versions at that time. Parentage testing was implemented using a custom R script following the method described by Hayes [31] that counted the number of opposing homozygous loci between offspring and potential parents. An animal was considered a parent if it was present at NDSU DREC in the breeding season that produced the offspring and had less than 1% opposing homozygous loci, given the number of genotyped loci passing quality filters. These results were manually reviewed for alignment with NDSU DREC mating records per animal. During parentage testing, initial marker statistics were generated to explore the NDSU DREC population for each analysis, which identified three females (two ADMX-I and one ADMX-II) as highly heterozygous (proportion of heterozygous genotypes greater than 0.90). This made parentage testing using opposing homozygous loci for five ADMX-II daughters challenging. Even so, none identified that a different genotyped dam was correct over her recorded dam. Through 643 parentage tests, only eight ADMX-II females had both parents genotyped but one parent was not confirmed (*n* = 6 for sires and 2 for dams) and a different genotyped parent of that same sex was not identified. These eight ADMX-II females and the three highly heterozygous females were removed from the current study to avoid biasing subsequent analyses. From the remaining parentage tests, only one individual had a sire of a different breed proven. No other errors were found; therefore, use of breed fractions based on mating records or confirmed parentage was assumed to be reasonable in creating population strata.

Genotype, family, and marker map data files for remaining individuals (*n* = 1140) were reformatted into PLINK .*ped* and .*map* file formats [24] to match WIDDE data. All data manipulation and formatting following parentage tests were completed in R software version 4.2.3 [29] using RStudio version 2023.03.03 [30] with *base* and *data.table* version 1.14.8 [32] package functions.

### 2.3. Population Strata

Based on mating records or known breeds from the two sources (NDSU DREC and WIDDE), animals in the genotyped population were first grouped based on their largest breed fraction (i.e., primary breed type, PBT). If animals were purebred (PBT = 1), then those animals were grouped by breed designation separately from admixed animals. For admixed cattle, influenced individuals (breed designation + I; 0.50 ≤ PBT < 1) must be at least 5% more than the next largest (secondary) breed fraction to avoid grouping true first cross (F_1_) individuals in influenced types. True F_1_ animals were created at DREC by mating known purebreds. These true F_1_ were grouped by type at the same level as PBT. There were three true F_1_ types in the genotyped population, including: F_1_ B × B, F_1_ B × C, and F_1_ C × C, where B refers to breeds AN, AR, HH, or SH and C refers to breeds GV or SM. The HH influence was only in F_1_ B × B individuals. The F_1_ B × C population was primarily B × GV (98.46%) since the F_1_ C × C cross was typically avoided and purebred SM at NDSU DREC were bulls. Creation of PBT resulted in 18 distinct groups of:

- 8 known purebred types (AD, AN, AR, GV, HH, LM, SH, SM),
- 7 known influenced types (ADI, ANI, ARI, GVI, LMI, SHI, SMI), and
- 3 F_1_ cross types.

**Figure 1:**
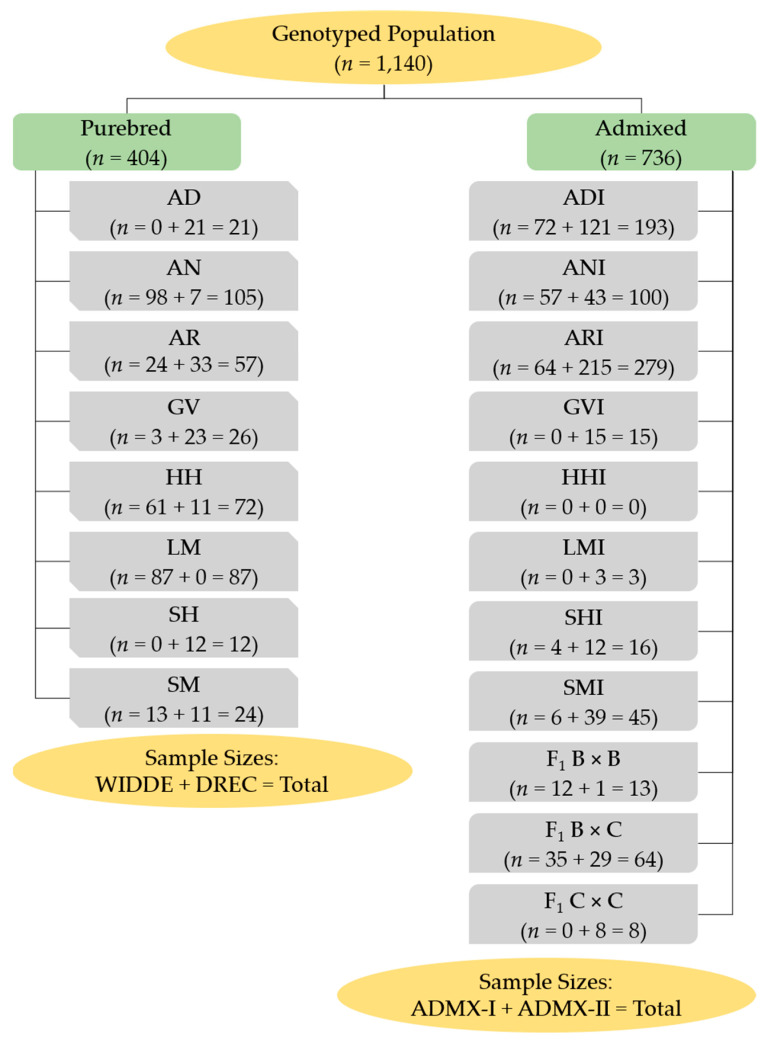
Sample sizes for the genotyped population grouped by purebred, admixed, and primary breed types (PBT) sub-populations after filtering. Purebred cattle (PBT = 1) included American Aberdeen (AD), Angus (AN), Red Angus (AR), Gelbvieh (GV), Hereford (HH), Limousin (LM), Shorthorn (SH), and Simmental (SM). Purebred cattle were sourced from Web-Interfaced next generation database dedicated to genetic Diversity Exploration (WIDDE) [23] and North Dakota State University Dickinson Research Extension Center (DREC) herd. Admixed cattle, sourced from DREC, included influenced (breed designation + I) and true first crosses (F_1_). Influenced cattle were individuals with PBT at least 0.50 but less than 1 and at least 5% more than the next largest (secondary) breed fraction. True F_1_ groups in the genotyped population included: F_1_ B × B, F_1_ B × C, and F_1_ C × C, where B refers to breeds AN, AR, HH, or SH and C refers to breeds GV or SM.

The PBT were the lowest strata besides individual. As influenced types have a large fraction of a known purebred type, the groups were reduced so that influenced types were absorbed into their respective purebred types (PT) as the next level of strata (*n* = 11 groups of AD, AN, AR, GV, HH, LM, SH, SM, and 3 F_1_ crosses). Lastly, an origin stratum was created as the highest level that grouped PT by country of origin, such that AD was labeled as origin 1 (Australian origin); AN, AR, HH, SH, and F_1_ B × B individuals as origin 2 (United Kingdom origin); GV, LM, SM, and F_1_ C × C individuals as origin 3 (Continental Europe origin); and F_1_ B × C as origin 4 (mixed origin). Figure 1 illustrates the genotyped population structure, PBT, and sample sizes.

### 2.4. Genotype Preparation for Diversity Analyses

Markers in the retained NDSU DREC genotyped population were assessed for CR and MAF using R package *snpStats* version 1.48.0 [33]. A total of 229 markers were removed due to 0% CR or 0% MAF. Markers from the WIDDE population were matched to the markers retained in the NDSU DREC population using Illumina name (original or synonym), where 26,794 markers were available in WIDDE and NDSU DREC populations. The two populations were merged using *snpStats* package with allele order checked and corrected before merging. Marker order was based on UCD 1.2 assembly coordinates. Quality checks on the combined population were assessed globally for CR and MAF. Markers identified with CR < 75% (*n* = 333) and MAF < 5% (*n* = 600) were removed, leaving 25,861 markers for subsequent quality checks. Following this, CR within each PBT was assessed for each marker, where markers that had all PBT with CR ≥ 95% were retained (*n* = 11,195 markers). Finally, individuals missing genotypes for a specific marker within each PBT were assigned the average genotype for that PBT to ensure all remaining markers were genotyped for all individuals. To avoid biasing imputed genotypes, imputation methods were not conducted due to the complex and unequally distributed nature of the genotyped population. The genotyped population’s files were formatted into PLINK .*ped* and .*map* file formats and exported following the same approach in R software described previously.

### 2.5. Genetic Diversity Analyses

It has been well documented that long-range linkage disequilibrium (LD) can influence both principal component and clustering analyses [11,34,35], but it remains unclear if LD present also impacts intrapopulation measures [36] given that neutral loci are not likely prevalent in cattle. Therefore, all analyses were approached with two genotype sets that either pruned or did not prune for LD. Linkage disequilibrium in the genotyped population was assessed in PLINK using the flag *--indep-pairwise 50 5 0.1* to identify markers in strong LD (i.e., pairwise genotypic correlation, r^2^ > 0.1). A secondary marker file (*n* = 4843 markers) was created by pruning high LD markers using the PLINK flag *--extract plink.prune.in*. Both marker files (unpruned and pruned) were then recoded into .*bed*, .*bim*, and .*fam* file formats for use in subsequent steps. The PLINK files were imported into R using the *plinkFile* package [37] and re-formatted into an *adegenet* version 2.1.10 package *genind* object [38,39] and *hierfstat* version 0.5-11 package data frame [40]. Both formats included four versions of (1) all animals (*n* = 18 PBT) using the unpruned marker set, (2) purebred only animals (*n* = 8 PBT) using the unpruned marker set, (3) all animals using the pruned marker set, and (4) purebred only animals using the pruned marker set. Two additional versions for *adegenet genind* format were made to set each animal’s genotype into an unphased haploid state (pruned marker set only) for (1) all animals and (2) purebred only animals to conduct analysis of molecular variance (AMOVA). Population labels for all formats were based on the lowest stratum (*n* = 8 or 18 PBT) but all strata (individual, PBT, PT, origin) were tied to *adegenet genind* formats in the *strata* tab for AMOVA.

#### 2.5.1. Intrapopulation Measures

Genetic diversity was characterized within populations using proportion of polymorphic loci (PPL), observed and within population gene diversity measures of heterozygosity (H_O_ and H_S_, respectively), allelic richness, and inbreeding coefficients (F_IS_). The PPL by PBT was calculated using the *snpStats* package *col.summary* function to find MAF, then the number of markers with MAF > 5% was counted and divided by the total number of markers. The remaining parameters were calculated using the *hierfstat* package *basic.stats* and *allelic.richness* functions. In all cases, both unpruned and pruned marker sets for (1) all animals and (2) purebred only animals were used. Using these estimates as seed values, each parameter was randomly sampled for 1000 iterations and the 2.5%, 50%, and 97.5% quantile values were extracted using base R *sample* and *quantile* functions of the *stats* package [29]. Lack of overlap between sub-populations for this 95% confidence interval supported differences in a given parameter.

#### 2.5.2. Interpopulation Measures

Genetic diversity was characterized across populations using genetic distance (D), fixation index (F_ST_), AMOVA, and discriminant analysis of principal components (DAPC). Both D and F_ST_ parameters were assessed using the unpruned and pruned marker sets in *hierfstat* package for (1) all animals and (2) purebred only animals given concerns noted about using unpruned markers in Li et al. [41] and Dementieva et al. [42]. The *genet.dist* function with options for standard genetic distance (D_S_), chord distance (D_CH_), and Latter’s F_ST_ as described in Takezaki and Nei [43] were used. Population specific and pairwise F_ST_ were estimated using *betas* (*n* = 100 bootstraps), *pairwise.neifst* (Nei’s F_ST_) [44], and *pairwise.WCfst* (WC F_ST_) [45] functions, respectively. Genetic distances and pairwise F_ST_ were assessed for fit as unrooted phylogenetic trees using neighbor-joining (*nj*) and *laddersize* functions of the *ape* version 5.7-1 R package [46], then tree distances were extracted using the *cophenetic* function of the *stats* R package and plotted against original distance with a linear regression line to mimic a quantile-quantile plot for fit. The distance measure with the best fit (highest alignment with linear regression line) was visualized using *base* R plotting functions.

All subsequent analyses utilized the pruned marker set since it is well established that LD can influence both principal component and clustering analyses [11,34,35]. For AMOVA analyses, the *poppr.amova* function in the *poppr* version 2.9.4 R package [47,48] was used with the haploid *genind* format using all relevant strata on (1) purebred PBT only (*n* = 3 strata levels) and (2) all PBT (*n* = 4 strata levels), which includes individual variance. Significance was determined using the *randtest* function of the *ade4,* version 1.7-22 R, package [49,50,51]. Due to computational time, the number of repetitions per *randtest* run were limited to 500 iterations since that would provide precision of the *p*-value at 0.02 [52]. Additional iterations were run if a particular level was less than 0.10 but not 0.05 to ensure adequate precision when assessing the null hypothesis.

Following recommendations by Thia [53], the number of clusters (*k*) expected was 7 (8 minus 1) for the purebred PBT only analysis, assuming each breed would create its own unique cluster, and 8 to 10 for all PBT analysis, depending on how the admixed populations separated. To determine if the number of expected clusters actually reflected the population, the *find.clusters* function of *adegenet* package was run with 10,000 iterations, *smoothNgoesup* selection criterion, and all axes from principal component analysis (PCA) retained (*n* = 398 for purebred only and 1134 for all animals). Following this, the cross-validation (*xvalDapc*) function of *adegenet* package was run initially at default settings to determine likely range of informative PCA axes to investigate further. Parameter settings were then changed to a smaller number of axes to investigate the specific range of interest for PCA axes with 1000 repetitions in parallel. This procedure automatically exports the DAPC run with the highest success rate and lowest mean square error. Clusters from DAPC analyses were investigated using the *scatter.dapc* function of *adegenet* package across all relevant axes. Final 3-dimensional visualizations were produced using the *rgl* version 1.0.1 R package [54]. To identify which linear discriminant axes (LDA) were separating AD, ADI, or both from other PBT, contributions of LDA retained were assessed by identifying the maximum distance between PBT present and the relative distance as a proportion for PBT (i.e., PBT the furthest apart would have proportions of zero and one). Variance contributions (loadings) per SNP marker were summed across genotypes per LDA to identify markers contributing in at least the top 2% per LDA and across LDA. Frequency differences among populations for identified SNP were found using the *seploc* function of the *adegenet* R package, then calculating the mean proportion per allele per PBT. Loading plots and allele frequencies were visualized using *base* R plotting procedures.

## 3. Results

**Figure 2:**
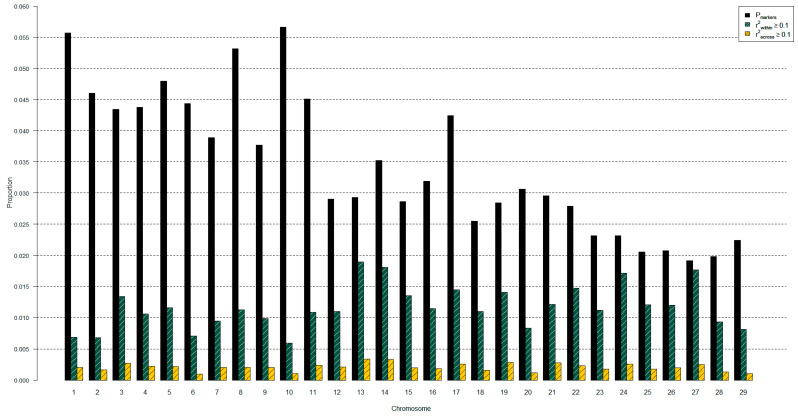
Attributes of genetic markers retained across populations of cattle by chromosome. Attributes include proportion of markers (P_markers_) out of total (*n* = 11,195) and proportion by chromosome for markers of pairwise genotypic correlation (r^2^; a measure of linkage disequilibrium) greater than 10% (1) within and (2) across chromosomes.

The proportion of SNP markers per chromosome ranged from 1.91% to 5.66% (Figure 2). Per chromosome, 0.59% to 1.90% of retained SNP markers had high LD within, and 0.10% to 0.34% had LD greater than 0.1 across chromosomes. The proportions used for Figure 2 are provided in Supplementary File S1. 

### 3.1. Average Population Measures

The overall population differentiation (D_est_ and F_ST_’) was larger when only purebred PBT were analyzed compared to all PBT, regardless of using unpruned or pruned marker sets (Table 1). Pruning the marker set resulted in decreased D_est_ and F_ST_’ for both analyses (purebred vs. all PBT; Table 1) resulting from the higher heterozygosity estimates (H_O_ and H_S_), particularly when admixed PBT were included.

### 3.2. Intrapopulation Measures

**Figure 3:**
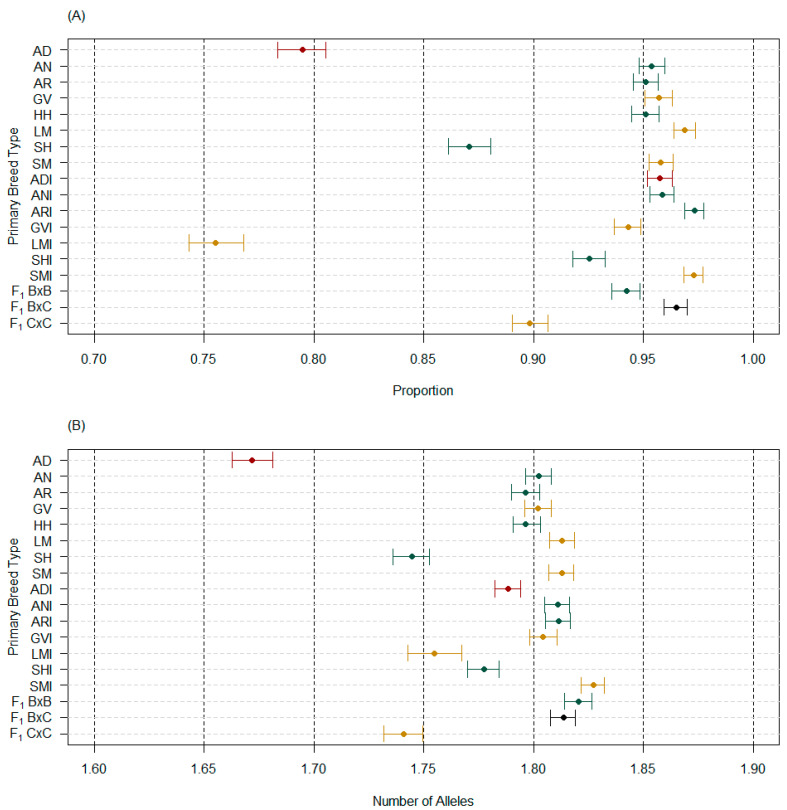
Median and 95% confidence interval estimates of all primary breed types (PBT) with the pruned marker set (*n* = 4843) for (**A**) proportion of polymorphic loci and (**B**) allelic richness (A_R_; bi-allelic markers, rarefraction = 6). Purebred cattle (PBT = 1) included American Aberdeen (AD), Angus (AN), Red Angus (AR), Gelbvieh (GV), Hereford (HH), Limousin (LM), Shorthorn (SH), and Simmental (SM). Admixed cattle included influenced (breed designation + I) and true first crosses (F_1_). Influenced cattle were individuals with PBT at least 0.50 but less than 1 and at least 5% more than the next largest (secondary) breed fraction. True F_1_ groups in the genotyped population included: F_1_ B × B, F_1_ B × C, and F_1_ C × C, where B refers to breeds AN, AR, HH, or SH and C refers to breeds GV or SM. The PBT are colored based on breed origin, where origin 1 (Australia) are dark red, origin 2 (United Kingdom) are green, origin 3 (Continental Europe) are dark yellow, and origin 4 (mixed origins) are black.

Bootstrapped median and 95% confidence intervals for PPL did not differ when considering purebred PBT on their own or with admixed PBT because the number of markers per analysis did not change. Changes between estimates from unpruned to pruned marker sets for purebred PBT ranged from 0.40% (AN) to 3.48% (SH), whereas admixed PBT ranged from 0.06% (SMI) to 2.74% (F_1_ C × C; Supplementary File S1). There were 13 of 18 PBT with lower PPL estimates using the pruned marker set compared to the unpruned marker set and the remaining 5 PBT had higher PPL estimates. The AD PBT was the second highest change in PPL estimate from unpruned to pruned marker sets, with a 3.30% increase using the pruned marker set. Considering the pruned marker set for all PBT, the median PPL within purebred PBT ranged from 0.7948 (AD) to 0.9688 (LM). Within admixed PBT, the median PPL ranged from 0.7553 (LMI) to 0.9734 (ARI). AD cattle had a considerably lower PPL for all purebred PBT (Figure 3A). Except for LMI, AD cattle also had a considerably lower PPL for all admixed PBT (Figure 3A). If more LMI samples were available (Figure 1), it is likely that AD cattle would be the lowest for PPL across all PBT. The only other purebred PBT with a median PPL less than 0.95 was SH. Of the admixed PBT, ADI was the fifth highest PPL of 10 admixed PBT (Figure 3A). 

The bootstrapped median and 95% confidence intervals for allelic richness, when considering purebred PBT on their own or with admixed PBT, differed due to rarefraction (*n* = 24 for purebred PBT only and *n* = 6 for all PBT), which accounted for an 8.07% to 10.46% decrease in median estimates when all PBT were included (Supplementary File S1). When considering the unpruned marker set compared to the pruned marker set, differences in allelic richness median estimates increased from 0.33% (AR) to 1.94% (SH) for the pruned marker set (Supplementary File S1). Although all PBT had less than a 2% change in estimates, pruning for LD did provide clarity between ADI cattle and other PBT. The median allelic richness estimate for ADI only increased by 0.79% when using the pruned marker set, whereas PBT with similar estimates in the unpruned marker set (AR, GV, HH, and LM) increased by 1.10% to 1.83% in the pruned marker set. In any case, AD cattle had substantially lower allelic richness than other PBT (Figure 3B). The ADI cattle were the fifth lowest admixed PBT of 10 admixed PBT for allelic richness (Figure 3B), following their ranking with PPL.

**Figure 4:**
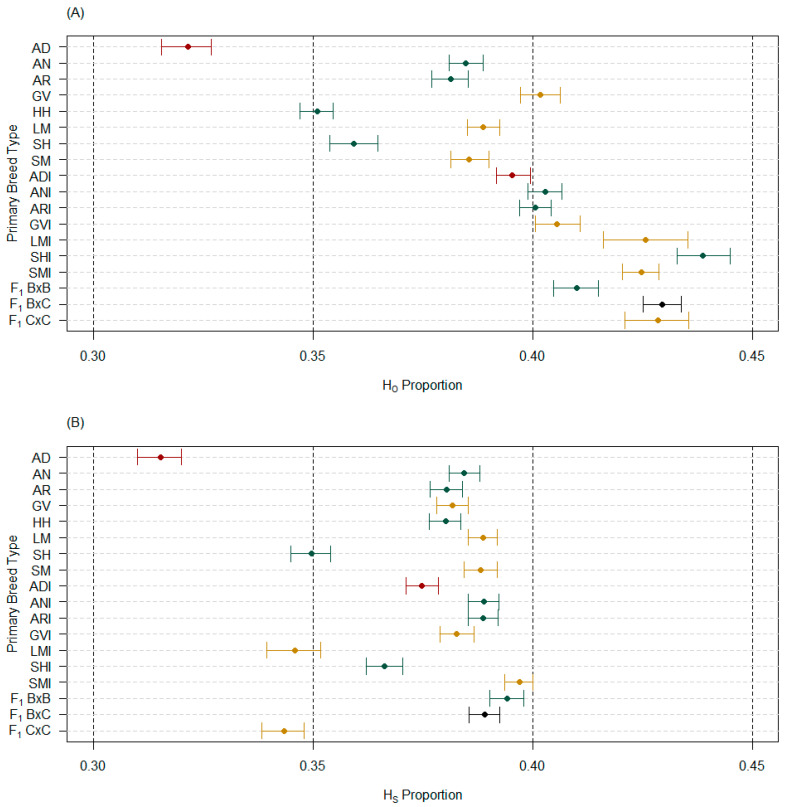
Median and 95% confidence interval estimates of all primary breed types (PBT) with the pruned marker set (*n* = 4843) for (**A**) observed heterozygosity (H_O_) and (**B**) within PBT gene diversity (H_S_). Purebred cattle (PBT = 1) included American Aberdeen (AD), Angus (AN), Red Angus (AR), Gelbvieh (GV), Hereford (HH), Limousin (LM), Shorthorn (SH), and Simmental (SM). Admixed cattle included influenced (breed designation + I) and true first crosses (F_1_). Influenced cattle were individuals with PBT at least 0.50 but less than 1 and at least 5% more than the next largest (secondary) breed fraction. True F_1_ groups in the genotyped population included: F_1_ B × B, F_1_ B × C, and F_1_ C × C, where B refers to breeds AN, AR, HH, or SH and C refers to breeds GV or SM. The PBT are colored based on breed origin, where origin 1 (Australia) are dark red, origin 2 (United Kingdom) are green, origin 3 (Continental Europe) are dark yellow, and origin 4 (mixed origins) are black.

The bootstrapped median and 95% confidence intervals for H_O_, when considering purebred PBT on their own or with admixed PBT, had minimal differences (largest was 0.0003), regardless of unpruned or pruned marker sets (Supplementary File S1). Within an analysis, comparing the unpruned and pruned marker sets resulted in H_O_ median estimate differences increasing by 0.92% (LMI) to 5.17% (SH) when using the pruned marker set (Supplementary File S1). Only three PBT (ANI, LMI, and F_1_ B × B) had less than a 2% change in median H_O_ estimates. Furthermore, AD cattle were consistently in the top three PBT for the largest change in estimates when considering unpruned to pruned marker sets. Changes observed in analyses (purebred only vs. all PBT) between unpruned and pruned marker sets were similar for within population gene diversity estimates (H_S_, Supplementary File S1) relative to changes observed in H_O_. In comparing PBT using the pruned marker set, AD cattle were consistently lower than any other PBT in H_O_ and H_S_ (Figure 4). Similar to allelic richness, ADI cattle had the lowest median H_O_ estimates of admixed PBT, where ADI confidence intervals only overlapped with ANI and ARI cattle (Figure 4A). The ADI cattle were not the lowest median H_S_ given that it accounts for the sample size of groups, but did separate more distinctly as lower H_S_ compared to ANI and ARI cattle (Figure 4B).

**Figure 5:**
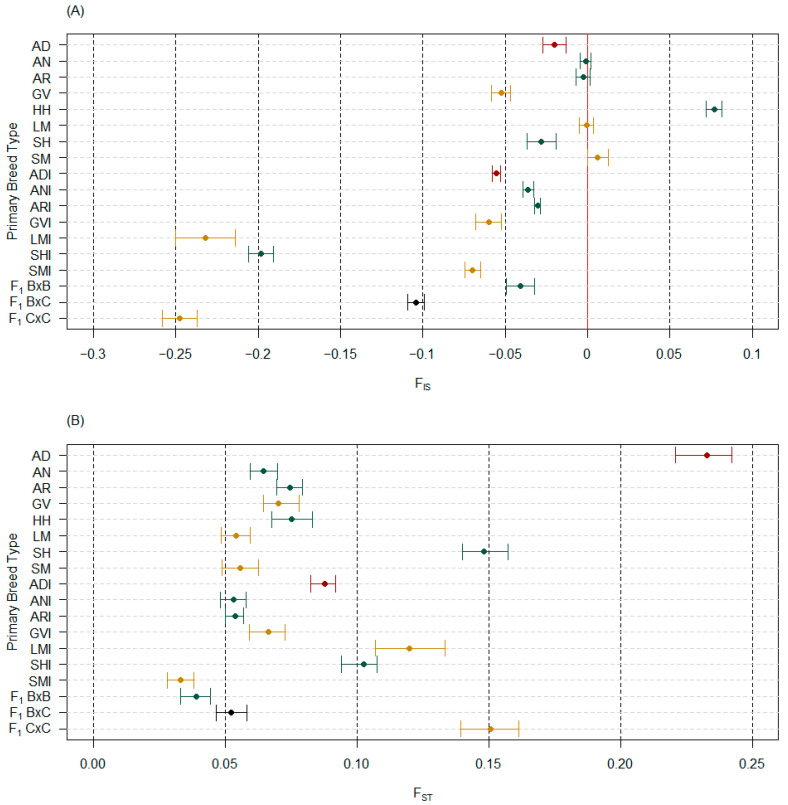
Median and 95% confidence interval estimates of all primary breed types (PBT) with the pruned marker set (*n* = 4843) for (**A**) inbreeding coefficients (F_IS_) and (**B**) fixation indexes (F_ST_). Expected levels of heterozygosity (F_IS_ = 0.00) are shown with a red line in (**A**). Purebred cattle (PBT = 1) included American Aberdeen (AD), Angus (AN), Red Angus (AR), Gelbvieh (GV), Hereford (HH), Limousin (LM), Shorthorn (SH), and Simmental (SM). Admixed cattle included influenced (breed designation + I) and true first crosses (F_1_). Influenced cattle were individuals with PBT at least 0.50 but less than 1 and at least 5% more than the next largest (secondary) breed fraction. True F_1_ groups in the genotyped population included: F_1_ B × B, F_1_ B × C, and F_1_ C × C, where B refers to breeds AN, AR, HH, or SH and C refers to breeds GV or SM. The PBT are colored based on breed origin, where origin 1 (Australia) are dark red, origin 2 (United Kingdom) are green, origin 3 (Continental Europe) are dark yellow, and origin 4 (mixed origins) are black.

The bootstrapped median and 95% confidence intervals for PBT inbreeding coefficients (F_IS_), when considering purebred PBT on their own or with admixed PBT, had minimal differences observed, similar to H_O_ and H_S_ (Supplementary File S1). Within an analysis, substantial differences between median estimates of F_IS_ from unpruned and pruned marker sets were observed, particularly for those populations estimated close to zero (i.e., at expected level of heterozygotes). In both cases, F_IS_ for AD cattle decreased by approximately 19%, indicating that the pruned marker set identified that AD cattle had an excess of heterozygotes at a higher amount than the unpruned marker set. Considering only the pruned marker set, four of eight purebred PBT (AN, AR, LM, and SM) had median estimates or 95% confidence intervals overlap at zero (Figure 5A). Only one purebred PBT had a median estimate and 95% confidence interval clearly above zero (HH), indicating a deficit of heterozygotes. The remaining three purebred PBT (AD, GV, and SH) had median estimates and 95% confidence intervals below zero, indicating an excess of heterozygotes. All admixed PBT were below zero, where ADI cattle were still indicating higher levels of excess in heterozygotes than ANI and ARI cattle (Figure 5A).

The bootstrapped median and 95% confidence intervals for the PBT fixation index (F_ST_), when considering purebred PBT on their own or with admixed PBT, had substantial differences observed (4.70% to 21.56%; Supplementary File S1) where estimates decreased when all PBT were included, regardless of using unpruned or pruned marker sets. Pruning for LD decreased median F_ST_ estimates for all PBT except three PBT (ANI, LMI, and F_1_ B × B), where changes ranged from 0.15% (GVI) to 31.13% (LM). Therefore, only results from pruned marker analyses are discussed for subsequent interpopulation measures. The median and 95% confidence intervals of F_ST_ estimates showed AD cattle were highly differentiated from other purebred PBT as well as admixed PBT (Figure 5B). The ADI cattle were the fourth highest F_ST_ in the admixed PBT estimated, including higher than ANI and ARI cattle (Figure 5B). 

### 3.3. Interpopulation Measures

#### 3.3.1. Genetic Distances and F_ST_

**Figure 6:**
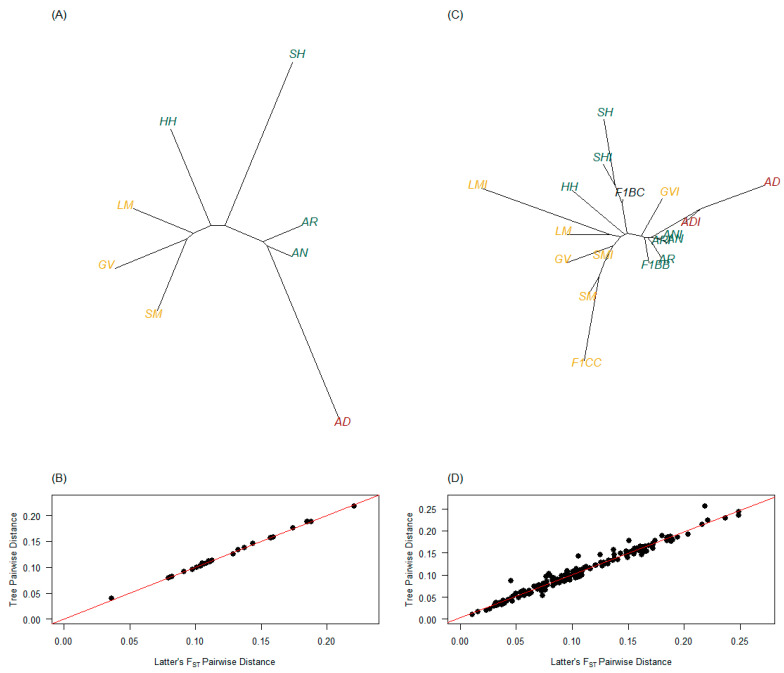
Phylogenetic trees with purebred primary breed types (PBT) only (**A**) or all PBT (**C**) and their fit (**B** and **D**, respectively) using unrooted neighbor-joining estimation methods based on Latter’s F_ST_ estimates. Purebred cattle (PBT = 1) included American Aberdeen (AD), Angus (AN), Red Angus (AR), Gelbvieh (GV), Hereford (HH), Limousin (LM), Shorthorn (SH), and Simmental (SM). Admixed cattle included influenced (breed designation + I) and true first crosses (F_1_). Influenced cattle were individuals with PBT at least 0.50 but less than 1 and at least 5% more than the next largest (secondary) breed fraction. True F_1_ groups in the genotyped population included: F_1_ B × B (F1BB), F_1_ B × C (F1BC), and F_1_ C × C (F1CC), where B refers to breeds AN, AR, HH, or SH and C refers to breeds GV or SM. The PBT are colored based on breed origin, where origin 1 (Australia) are dark red, origin 2 (United Kingdom) are green, origin 3 (Continental Europe) are dark yellow, and origin 4 (mixed origins) are black.

For purebred only PBT, all measures of pairwise genetic distance performed similarly in constructing the phylogenetic tree using the unrooted neighbor-joining method (Supplementary Files S2; Figure 6A,B for Latter’s F_ST_). The purebred PBT grouped in clusters similar to their country of origin, where AD cattle were highly differentiated from other purebred PBT (Figure 6A). When considering all PBT, measures of pairwise genetic distance differed in how well the estimates mirrored tree distance estimates (Supplementary File S2). When using the unrooted neighbor-joining method, tree distances based on Latter’s F_ST_ resulted in the best alignment to original estimates (Supplementary File S2, Figure 6D). Therefore, the results of the genetic distance and phylogenetic tree are discussed relative to Latter’s F_ST_ estimates. Purebred PBT with all PBT included followed branching, observed when only purebred PBT were analyzed (Figure 6A,C). Furthermore, admixed PBT branching aligned with expectations. For example, 47 of 64 F_1_ B × C (73.4%) were SH × GV crosses, providing support for the alignment of F_1_ B × C on the SH branch and between GV and GVI. The same argument can be made for F_1_ C × C (*n* = 8) since they were all SM × GV crosses, leading to the connection of GV, SM, and their admixed PBT being on the same branch. Furthermore, all GVI (*n* = 15) had AN or AR as their secondary breed type, providing support for their alignment close to those respective PBT branches. Lastly, 137 of 193 ADI cattle (71.0%) had AN or AR as their secondary breed type, also supporting their pull away from AD towards those respective PBT branches.

#### 3.3.2. Analysis of Molecular Variance

**Figure 7:**
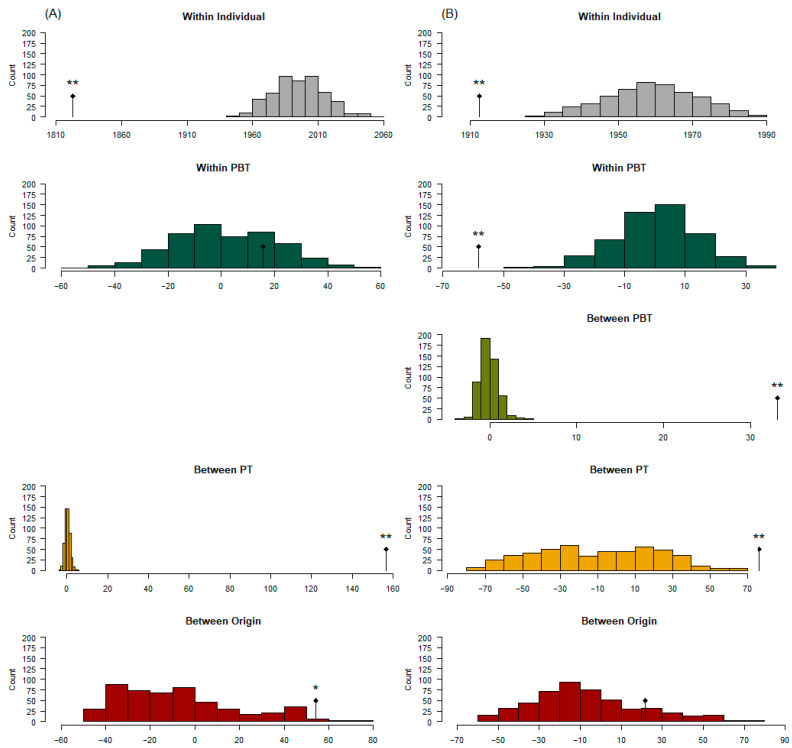
Analysis of molecular variance considering (**A**) purebred only (*n* = 8) and (**B**) all (*n* = 18) primary breed types (PBT). Corresponding colors between (**A**,**B**) are the same level of population strata. Purebred cattle (PBT = 1) included American Aberdeen (AD), Angus (AN), Red Angus (AR), Gelbvieh (GV), Hereford (HH), Limousin (LM), Shorthorn (SH), and Simmental (SM). Admixed cattle included influenced (breed designation + I) and true first crosses (F_1_). Influenced cattle were individuals with PBT at least 0.50 but less than 1 and at least 5% more than the next largest (secondary) breed fraction. True F_1_ groups in the genotyped population included: F_1_ B × B, F_1_ B × C, and F_1_ C × C, where B refers to breeds AN, AR, HH, or SH and C refers to breeds GV or SM. Purebred types (PT) stratum (*n* = 8 or 11 for (**A**) or (**B**), respectively) reduced influenced individuals into their respective purebred PBT except for F_1_ crosses. Origin stratum grouped PT by country of origin such that AD was labeled as origin 1 (Australia); AN, AR, HH, SH, and F_1_ B × B individuals as origin 2 (United Kingdom); GV, LM, SM, and F_1_ C × C individuals as origin 3 (Continental Europe); and F_1_ B × C as origin 4 (mixed origin). Significance of the observed variance is indicated as * (*p* < 0.05) and ** (*p* < 0.005) above the observed variance marker (black segmented diamond).

Figure 7 identifies observed variance estimates within and between different strata as well as their significance based on 500 permutations (*p*-value ± 0.02). The AMOVA with purebred only PBT was completed in approximately 33 min for 500 permutations and 9 h for 1000 permutations using a 64-bit Windows 10 operating system with 64 GB RAM and an Intel^®^ Xeon^®^ W-2245 8-core, 16-thread 3.91 GHz base frequency processor. *p*-values for the 1000 permutations only decreased by 0.0012, on average compared with 500 permutations. For example, the between origin levels of purebred only PBT resulted in a *p*-value at 500 permutations of 0.01996 ± 0.02236 and at 1000 permutations of 0.01798 ± 0.01581. All other tests with 500 permutations for purebred only PBT analyses resulted in a *p*-value = 0.001996 (±0.02236). The AMOVA with all PBT was completed in 2.1 d for 500 permutations. The origin stratum *p*-value was above the 0.10 threshold (*p*-value = 0.1597) and would not warrant extended computational time to prove whether it would reject the null hypothesis. All other tests for the analysis resulted in a *p*-value = 0.001996 (±0.02236); therefore, additional permutations were not completed. When considering only purebreds as part of the strata (Figure 6A), origins (*n* = 4) accounted for 2.65%, between PT within origins (*n* = 8) accounted for 7.65%, between individuals within PBT (*n* = 12 to 105 individuals of 8 PT, where PBT = PT) accounted for 0.76%, and within individuals (diploid) accounted for 88.94% of the genetic variation. When including all PBT as part of the strata (Figure 6B), origins accounted for 1.10%, between PT within origins (*n* = 11) accounted for 3.84%, between PBT within PT (*n* = 18) accounted for 1.67%, between individuals within PBT (*n* = 3 to 279 individuals of 18 PBT) accounted for −2.94% of the variation, and within individuals accounted for 96.32% of the genetic variation.

#### 3.3.3. Discriminant Analysis of Principal Components

**Figure 8:**
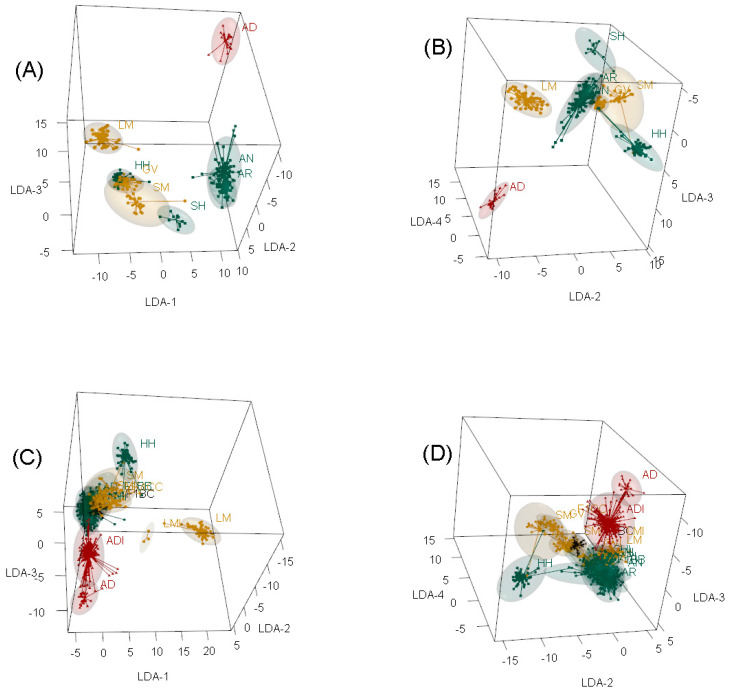
Three-dimensional visualization of the first four linear discriminant axes (LDA) from discriminant analysis of principal components for purebred only (**A**,**B**) and all (**C**,**D**) primary breed types (PBT). Purebred cattle (PBT = 1) included American Aberdeen (AD), Angus (AN), Red Angus (AR), Gelbvieh (GV), Hereford (HH), Limousin (LM), Shorthorn (SH), and Simmental (SM). Admixed cattle included influenced (breed designation + I) and true first crosses (F_1_). Influenced cattle were individuals with PBT at least 0.50 but less than 1 and at least 5% more than the next largest (secondary) breed fraction. True F_1_ groups in the genotyped population included: F_1_ B × B (F1BB), F_1_ B × C (F1BC), and F_1_ C × C (F1CC), where B refers to breeds AN, AR, HH, or SH and C refers to breeds GV or SM. The PBT are colored based on breed origin, where origin 1 (Australia) are dark red, origin 2 (United Kingdom) are green, origin 3 (Continental Europe) are dark yellow, and origin 4 (mixed origins) are black. The 95% confidence ellipses are shaded regions around a given PBT.

For the purebred only PBT, PCA identified that the first seven axes (expected *k*) accounted for 17.44% of the variation. Using the *find*.*clusters* algorithm indicated that *k* = 6 was ideal (BIC = 2796.13; 16.34% of 17.44%), where *k* = 5 and 7 BIC differed by only 0.056 and 0.401, respectively. A cross-validation DAPC analysis found that the number of principal components (PC) with the lowest mean square error (MSE) was 12, with the first 23 PC having the highest mean success. The cross-validation DAPC retained the first 12 PC and the first seven LDA, which resulted in 22.20% of conserved variance. The maximum distance between population average coordinates ranged from 5.43 units (LDA-7) to 21.06 units (LDA-1). Using LDA-1, AD and LM were the furthest apart (21.06 units), with HH and GV being 77.14% and 75.54% of that distance from AD also. The AN and AR PBT were closest to AD using LDA-1, at 7.32% and 9.68% of the distance between AD and LM. Using LDA-2, AD separated the most from HH (16.13 units), followed closely by SH (93.37%) and SM (94.62%). LDA-2 separated AN (51.37%) and AR (55.51%) from AD more than LDA-1 and retained GV at 80.31% of that distance. Using LDA-3, AD separated the most from SH (16.11 units), followed closely by AR (97.18%), then LM (87.51%), AN (86.42%), SM (82.42%), GV (75.71%), and HH (58.12%). Starting with LDA-4, axis coordinates separated other purebred PBT with each other rather than AD. When visualized, the first three LDA identified four primary clusters (Figure 8A, left to right):

- Group 1: LM;
- Group 2: GV, HH, SH, and SM;
- Group 3: AD; and
- Group 4: AN and AR.

**Figure 9:**
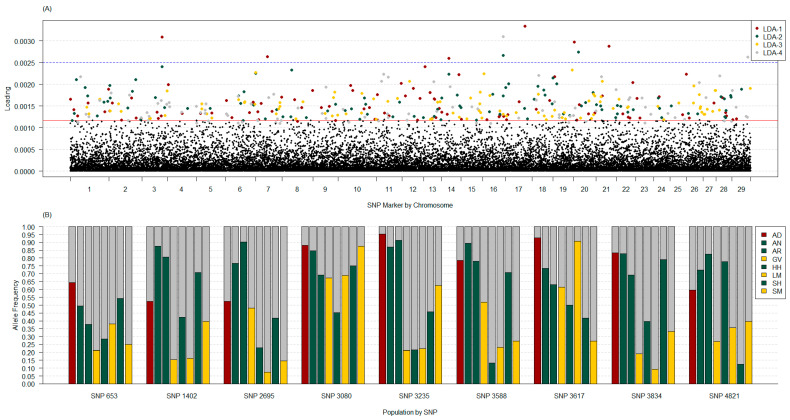
Loading plot of variable contribution per SNP marker by chromosome across four discriminant analyses of principal component axes (LDA; part (**A**)) and allele frequency by purebred primary breed types (PBT) for nine markers with variable contribution over 0.0025 (**B**). Markers in the top 2% per LDA are colored by axes with the average threshold across axes as a solid red line (**A**). Markers above the 0.0025 threshold (blue line) in (**A**) are presented as proportion of allele 1 (colored) and allele 2 (grey) by population in (**B**). Colors reflect breed country of origin. Purebred PBT included American Aberdeen (AD, dark red), Angus (AN, green), Red Angus (AR, green), Gelbvieh (GV, yellow), Hereford (HH, green), Limousin (LM, yellow), Shorthorn (SH, green), and Simmental (SM, yellow). The nine SNP markers include: 653 (ARS-BFGL-NGS-1038: BTA 3 104.37 Mb), 1402 (ARS-BFGL-NGS-12557: BTA 7 45.67 Mb), 2695(ARS-BFGL-NGS-110427: BTA 14 22.67 Mb), 3080 (ARS-BFGL-NGS-56: BTA 16 76.44 Mb), 3235 (ARS-BFGL-NGS-118636: BTA 17 58.11 Mb), 3588 (ARS-BFGL-NGS-34277: BTA 20 2.06 Mb), 3617 (Hapmap-42340-BTA-84327: BTA 20 15.75 Mb), 3834 (ARS-BFGL-NGS-42945: BTA 21 44.60 Mb), and 4821 (US-IF-ASA-1370: BTA 29 44.42 Mb).

These four clusters were still visible until LDA-4 became the primary axis (Figure 8A,B, and Supplementary File S3A,B). Even so, AD remained distinctly apart from other purebred PBT until LDA-5 was the secondary axis (Figure 8 and Supplementary File S3). When considering the variable contribution per SNP marker, the top 98% across LDA per allele had a loading of at least 0.00053. After summing allele loadings per marker, the top 98% across LDA were at least 0.00109. Because LDA-1 to LDA-4 provided the differentiation of AD from other purebred PBT, loading plots across these four LDA were investigated (Figure 9A). Based on these marker loadings, there were 10 occurrences of loadings over 0.0025, where six had high loadings for LDA-1, two for LDA-2, and two for LDA-4. A single SNP for LDA-2 and LDA-4 on BTA 16 was in this group, leaving a total of nine SNP covering these 10 occurrences. The nine SNP were located on BTA 3, 7, 14, 16, 17, 20, 21, and 29. Frequency differences among purebred PBT of these nine SNP illustrated some population differences (Figure 9B). Six of the nine SNP had locus F_ST_ and F_ST_’ in the top 95% of the pruned marker set (*n* = 243), with F_ST_ and F_ST_’ greater than 0.2063 and 0.2290, respectively. Even so, the locus F_ST_ estimates did not always translate to high loads in DAPC (Supplementary File S4).

**Figure 10:**
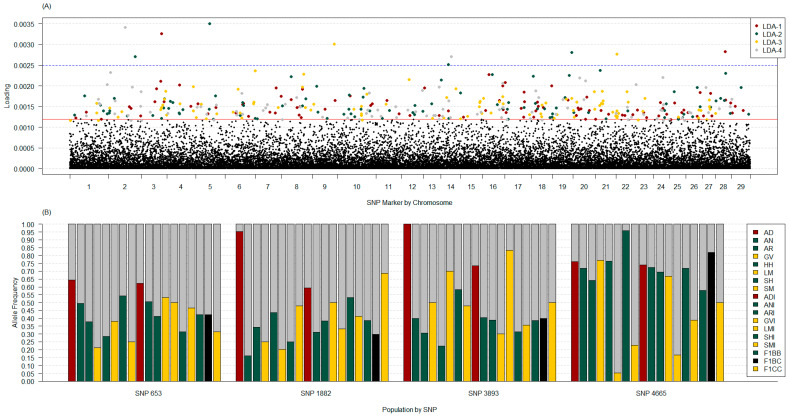
Loading plot of variable contribution per SNP marker by chromosome across four discriminant analyses of principal component axes (LDA; part (**A**)) and allele frequency by primary breed types (PBT) for four markers with variable contribution over 0.0025 on LDA-1 and LDA-3 (**B**). Markers in the top 2% per LDA are colored by axes with the average threshold across axes as a solid red line (**A**). Specified markers above the 0.0025 threshold (blue line) in (**A**) are presented as proportion of allele 1 (colored) and allele 2 (grey) by PBT in (**B**). Purebred cattle (PBT = 1) included American Aberdeen (AD), Angus (AN), Red Angus (AR), Gelbvieh (GV), Hereford (HH), Limousin (LM), Shorthorn (SH), and Simmental (SM). Admixed cattle included influenced (breed designation + I) and true first crosses (F_1_). Influenced cattle were individuals with PBT at least 0.50 but less than 1 and at least 5% more than the next largest (secondary) breed fraction. True F_1_ groups in the genotyped population included: F_1_ B × B (F1BB), F_1_ B × C (F1BC), and F_1_ C × C (F1CC), where B refers to breeds AN, AR, HH, or SH and C refers to breeds GV or SM. The PBT are colored based on breed origin, where origin 1 (Australia) are dark red, origin 2 (United Kingdom) are green, origin 3 (Continental Europe) are dark yellow, and origin 4 (mixed origins) are black. The four SNP markers include: 653 (ARS-BFGL-NGS-1038: BTA 3 104.37 Mb), 1882 (Hapmap-38533-BTA-84943: BTA 9 96.18 Mb), 3893 (ARS-BFGL-NGS-103597: BTA 22 51.40 Mb), and 4665 (ARS-BFGL-NGS-111706: 28 33.87 Mb).

When all PBT were included, PCA identified that the first 10 axes (expected *k*) accounted for 15.35% of the variation, whereas the first seventeen axes (number of PBT minus one) accounted for 19.61%. Using the *find*.*clusters* algorithm indicated that *k* = 10 was ideal (BIC = 7803.98), where *k* = 8 and 11 BIC differed by less than one unit. The cross-validation DAPC analysis found that the number of PC with the lowest MSE and highest mean success was 93. The cross-validation DAPC retained the first 93 PC and the first 17 LDA functions. This resulted in 43.70% of conserved variance. The maximum distance between the population average coordinates ranged from 1.76 units (LDA-17) to 24.07 units (LDA-1), where LDA-5 was the only LDA under 13 units in the first seven LDA. All other LDA had distances less than 9, including LDA-5. Using LDA-1, AD and LM were the largest separation (24.07 units), with the only other group over 50% of that distance being LMI (52.69%). At 20.86% and 24.41%, GV and HH were the next largest separation from AD, which followed separations seen in the purebred only analysis for LDA-1. The ADI PBT was the closest to AD at only 4.19% of the maximum distance, followed by SH (5.22%), AN (5.07%), AR (6.36%), ANI (7.08%), and ARI (7.08%). The remaining groups were between 8% and 15% of the maximum distance. Using LDA-2, AN and HH were the furthest apart (14.80 units), where GV (43.34%) and SM (55.43%) were the next furthest PBT from AN. The AD and LM PBT were closest to AN at 5.74% and 3.29% of the maximum distance, respectively. The ADI, ANI, and ARI PBT grouped approximately 12% from AN. Using LDA-3 provided the largest separation of AD from all other PBT. The maximum distance was between AD and AR at 14.15 units, where all PBT were at least 61.58% (LMI) of that distance from AD, except ADI (38.98%). The remaining axes separated other PBT from the main cluster, but LDA-4 AD and ADI were found within the main cluster (Figure 8C,D, Supplementary File S3C,D). The loading plot identified four SNP relevant for LDA-1 and LDA-3, following the same thresholds as the purebred only PBT analysis (Figure 10). One SNP, ARS-BFGL-NGS-1038 (number 653) on BTA 3 at 104.37 Mb, had high loading for both DAPC analyses (purebred only vs. all). None of the four SNP on LDA-1 and LDA-3 with high loads had high locus F_ST_ estimates (Supplementary File S4).

## 4. Discussion

Several breed registries in the USA allow crossbreds with a high fraction of that respective breed to register (i.e., graded up individuals) as purebreds when a desired fraction is proven. These registries often use codes to distinguish the graded-up animals from known full-bloods (i.e., animals tracing to foundational breed animals). Breeds used in this study that allow graded up individuals to register include AD, AR, GV, LM, SH, and SM [55,56,57,58,59,60]. Although the USA AN registry does not allow graded up individuals to register within their association (i.e., a closed registry), both AN and AR registries can be influenced by each other since they are both members of the World Angus Secretariat [61]. The USA AN and HH registries remain one of the few breed associations in which graded up individuals are not allowed [62,63]. Given that many of the breeds used in this study allow grading up or influence each other to some extent, an inherent level of migration was expected yet challenging to quantify in the samples used, particularly WIDDE samples. Therefore, there was concern initially on whether these breed samples would clearly separate themselves from each other, even when known purebreds were considered without admixed individuals. The clustering of AN and AR (Group 4) and GV, HH, SH, and SM (Group 2) when considering purebred only PBT (Figure 8 and Supplementary File S3) was, therefore, not surprising. Thia [53] studied simulated populations based on rates of migration and showed that high migration rates will cause issues in DAPC separating known or expected sub-populations well. This supports the inherent migration present in the current study, which was at moderate levels in the purebred PBT only DAPC and high levels in the DAPC using all PBT that included related, admixed individuals to most purebred PBT (Figure 8 and Supplementary File S3). Even so, the distinct differences seen in AD and their influenced admixed offspring (ADI) were evident, further supporting that AD cattle are genomically distinct from the other breeds, including their closest relatives AN and AR. 

The current study is the first to genomically characterize AD cattle for basic genetic diversity measures, as well as comparing it to other breeds of cattle. The AD cattle used in this study were primarily sourced from bulls produced by the Effertz EZ Ranch in ND (18 of 21 samples), who contributed largely to the establishment of the AD breed in the USA [20]. Of the 21 AD samples, only 2 had unknown registry status (registration data were lost) and the remaining 19 samples were registered full-bloods (i.e., full-blood male, FM, registration designation—tracing to foundational, registered Australian low-line cattle). Therefore, these samples provide a realistic perspective of the genetic diversity within AD cattle as well as compared to other breeds used in this study (e.g., Figure 3, Figure 4 and Figure 5 vs. Table 1). The phylogenetic trees clearly show that AD and ADI cattle separated from other PBT (Figure 6), even though ADI cattle pull towards PBT influencing them (i.e., AN and AR were common secondary breed fractions).

The SNP markers used in the current study were based on the Illumina BovineHD, Illumina BovineSNP50 version 1, GGP150K, and GGP100K panels [23,26]. The Illumina BovineHD panel is the most comprehensive and has the highest relationship of shared markers to the other three panels. Even so, the BovineSNP50 panel does not share as many common markers as the GGP panels, and the proprietary markers included on the GGP provide further differences from both Illumina panels [64]. Selecting markers present across all of these panels given the available samples likely reduced ascertainment bias to some degree for AD that may have otherwise occurred if all animals were genotyped on the same panel. Given the available documentation, it does not appear that AD cattle have been used for any genetic marker panel development to date [64,65].

Furthermore, SNP markers used in the study had high call rates within and across PBT and avoided low MAF globally; therefore, it is not surprising that the majority (14 of 18) of the PBT expressed high PPL (i.e., greater than 90%; Figure 3A). This, in turn, made it possible to see high allelic richness (i.e., greater than 1.5 of 2; Figure 3B) and moderate levels of gene diversity in terms of heterozygotes (Figure 4). Even so, AD cattle were consistently the lowest in these intrapopulation measures and significantly lower than breeds that could have similar origins (e.g., AN, AR, HH, SH). In fact, of the 2594 markers with an MAF below 0.05 for AD using the unpruned marker set, 1521 (58.64%) had an MAF of zero. The only other purebred PBT similar was SH (55.84% of 1789 markers with an MAF less than 0.05 were zero), which matches why its estimates of intrapopulation measures were also low compared to other populations. Other purebred PBT ranged from 541 (LM) to 744 (HH) markers below an MAF of 0.05 and 10.71% (AN) to 23.73% (SM) of those having an MAF equal to zero using the unpruned marker set. Pruning markers did reduce the number of markers below an MAF of 0.05, but did not change the relationship of percentage equal to zero for purebred PBT (e.g., 994 markers below an MAF 0.05 and 56.74% equal to zero for AD). 

Both AD and SH PBT samples were sourced from the NDSU DREC. There were no possible AD samples through WIDDE and all SH samples through WIDDE failed call rate control, even when set as low as 90%. The failure of WIDDE SH samples was related to the Illumina BovineSNP50 version 1 data available, where over 10% of those markers matching the other panels selected were not called for all SH samples listed. Given this, both AD and SH PBT in this study have less diversity than other PBT included. Hanwoo cattle in a study of Ethiopian, African, and Asian cattle breeds were also found to be low in polymorphic loci and estimates of heterozygosity [6]. In their study, the authors attributed this to SNP ascertainment bias since Hanwoo was the only taurine-based breed on the marker panels used and the panels were developed on European breeds. Edea et al. [6] pruned for LD to remove ascertainment bias in their study. Although ascertainment bias may be influencing estimates in the current study (i.e., a reason ADI cattle separated better after LD pruning), it is not likely to be the primary driver behind the lower estimates in AD and SH cattle simply because estimates after pruning for LD remained considerably lower for these two PBT than the other PBT included. For this study, effective population size (i.e., available lineages) and selection are stronger reasons for the lower levels of intrapopulation diversity observed in these two breeds. The current study also showed that LD pruning can impact intrapopulation measure estimates across breeds used, which may be due to sampling bias of animals [36] or genetic markers [6]. As indicated earlier, it is unlikely that ascertainment bias of genetic markers is a large contributor in the current study, although sample bias in available lineages remains a strong rationale for why LD pruning was useful in intrapopulation measures. Given this, genetic diversity studies in livestock moving forward likely need to prune for LD in all parameters estimated. Even so, the reasons for LD pruning in intrapopulation measures of the current study need to be elucidated further.

The ADI cattle were often intermediate to other admixed PBT for genetic diversity parameters. In admixed individuals, most (93.61%) had two to three breeds influencing them (ordered by largest to smallest breed fractions), with the highest number being six breeds (two individuals). Within admixed individuals, ADI made up 26.22% of the population, with secondary breeds consisting of AN (25.39%), AR (29.53%), GV (6.22%), HH (1.55%), LM (2.59%), SH (12.44%), SM (12.95%), and unknown ancestry (9.32%). In addition, there were 167 (22.69%) admixed individuals with some level of AD influence (second to fourth breed only). When AD was the secondary breed (91.02% of 167 individuals), fractions ranged from 1/16 to 3/8 and AR as the primary breed was the most common (88.16%), followed by AN (10.53%) and LM (1.32%). As the third breed (8.38% of 167), fractions ranged from 1/16 to 3/16 and AR was again the most common primary breed (42.86%), followed by AN (35.71%), LM (7.14%), and SM (14.29%). The single animal with AD as the fourth breed (1/16 fraction) again had AR as the primary breed. This breakdown of AD influence in the admixed population was not characterized prior to the study commencing and explains why the ADI population often resulted in intermediate estimates to other admixed PBT. The lower intrapopulation estimates of AD combined with the majority of animals with some level of AD influence paired with AN and AR would not result in having high levels of heterozygosity compared to other possible admixed types.

A basic attribute of domestic livestock is selection pressure placed on a given type to enhance the desired production attribute(s), thereby creating a breed known for specific production as well as population sub-structures within and across breed types. With increased numbers of cattle breed associations in the USA allowing graded up individuals to register as a form of purebred cattle, including AD, care should be taken in conserving diversity and effective population sizes relative to full-blood lines if future needs, such as economic and/or ecological fluctuations [66], arise. The sheer numbers of registered animals assist some breeds in maintaining adequate effective population sizes, as demonstrated by Márquez et al. [67], but this does not provide evidence of full-blood diversity and conservation. In the event that full-blood diversity is low (i.e., effective population sizes are below desired levels), identifying purebred lines, including graded up individuals, carrying ancestral genomes would be ideal. This could be done by identifying the precise breed fractions of an admixed individual, such as those that could be produced in admixture analyses [68]. Even so, if the true nature of admixture in a given population is not known or the process may involve many sources of admixture with related individuals, such as the NDSU DREC population in the current study, traditional admixture analyses will result in false interpretations [68]. It is evident from the current study that using an output such as DAPC is challenging when heavy admixture is present due to higher levels of migration and relatedness of breeds [53,69]. The cross-validated DAPC when including all PBT could only assign individuals accurately to their PBT 83.20% of the time, which aligns with the lack of separation of those admixed PBT in Figure 8. Given this, a traditional approach, such as an admixture analysis, would also struggle to describe a clear proportion of each breed influencing a given animal. Therefore, other means of separating regions of individual genomes by breed of origin are necessary. 

Given that selection places a critical role in the development of most domestic cattle breeds, identifying signatures of selections [70], such as runs of homozygosity and LD decay [71,72,73,74], or potentially copy number variations [75] could provide snapshots of the genome to accurately assign the breed of origin in admixed and graded up populations. Lawson et al. [68] illustrated a similar concept using chromosome painting, but implementation of this or other approaches for breed assignment remain inconsistent and problematic [76,77,78,79,80,81,82,83,84]. Characterizing these attributes in full-blood AD compared to other common beef breeds as well as assigning genomic breed fractions within the NDSU DREC admixed population could provide an opportunity to explore the diversity and feasibility of these methods further in a species where intense selection pressures dominate the evolution of allele and genotype frequencies.

## 5. Conclusions

This study proves that AD cattle are genomically distinct from common USA beef breeds. Through this study, the presence of admixture within USA purebreds was also shown given the challenge of accurately assigning individuals to the correct strata, particularly when admixed individuals were present in the population analyzed. Given the by-laws of many USA breed associations, this admixture in purebreds was not surprising. Rather, it emphasizes the increasing challenge of maintaining genetic diversity, if that is desired in the future, for those breeds that allow grading up processes, such as AD. As demonstrated in this study, this admixture in purebreds also highlights the challenge in identifying the accurate proportions that an admixed individual may possess. This approach is increasingly popular in livestock as a traceability measure; however, outcomes from the admixed populations analyzed in this study show that continual introgression in breed associations may make that option infeasible in cattle. Additional research on other measures of genetic diversity or differentiating populations based on genomic data will need to be explored with the study’s current dataset to determine if any methodologies can accurately show the breed proportions of admixed animals in this study.

## Figures and Tables

**Table 1:** Basic population diversity statistics given primary breed type (PBT) populations and marker sets used ^1^.

	Purebred PBT			All PBT		
Estimate ^2^	Unpruned	Pruned	Change, % ^3^	Unpruned	Pruned	Change, % ^3^
H_O_	0.3565	0.3718	4.12	0.3833	0.3965	3.33
H_S_	0.3560	0.3713	4.12	0.3629	0.3756	3.38
H_T_	0.4006	0.4118	2.72	0.3986	0.4090	2.54
D_ST_	0.0449	0.0405	−9.80	0.0357	0.0334	−6.44
F_ST_	0.1119	0.0983	12.15	0.0897	0.0816	−9.03
H_T_’	0.4073	0.4175	2.44	0.4007	0.4109	2.48
D_ST_’	0.0513	0.0463	−9.75	0.0378	0.0353	−6.61
F_ST_’	0.1259	0.1108	−11.99	0.0944	0.0859	−9.00
F_IS_	−0.0012	−0.0014	16.67	−0.0564	−0.0556	−1.44
D_est_	0.0796	0.0736	−7.54	0.0594	0.0566	−4.71

^1^ Purebred cattle (PBT = 1) included American Aberdeen, Angus, Red Angus, Gelbvieh, Hereford, Limousin, Shorthorn, and Simmental. Admixed cattle included influenced and true first crosses. Purebred PBT only analyses included 8 sub-populations whereas all PBT analyses included 18 sub-populations. Unpruned marker set (*n* = 11,195) was pruned for markers exhibiting linkage disequilibrium (r^2^) over 0.1, leaving the pruned marker set with 4843 markers. ^2^ Parameter estimates include: observed heterozygosity (H_O_), within population gene diversity (H_S_), overall gene diversity (H_T_), interpopulation gene diversity (D_ST_), fixation index (F_ST_), inbreeding coefficient (F_IS_), corrected estimates for removing population *k* comparison to itself (H_T_’, D_ST_’, and F_ST_’), and overall population differentiation (D_est_). ^3^ The percent of change was calculated as pruned marker set estimate minus unpruned marker set estimate divided by the maximum estimate for pruned or unpruned marker sets times 100.

## Data Availability

Cattle genotypes from WIDDE are publicly available at http://widde.toulouse.inra.fr/widde/ (accessed on 10 August 2023). Raw or processed genotypes of DREC cattle can be made available by request to the corresponding author. Data summarized in this publication along with corresponding custom R scripts, functions, and RData files generated are available at https://doi.org/10.5281/zenodo.8235249 (accessed on 10 August 2023).

## Supplementary Materials

The following supporting information can be downloaded at: https://www.mdpi.com/article/10.3390/genes14101842/s1, File S1: Spreadsheets of Figure 2, allelic richness (A_R_), observed heterozygosity (H_O_), gene diversity (H_S_), population inbreeding coefficients (F_IS_), and population differentiation (F_ST_) estimates when using pruned or unpruned marker sets; File S2: Spreadsheets comparing genetic distance measure estimates and their respective neighbor-joining distances; File S3: Three-dimensional visualization of the linear discriminant axes (LDA) three to six from discriminant analysis of principal components for purebred only (A,B) and all (C,D) primary breed types (PBT). Purebred cattle (PBT = 1) included American Aberdeen (AD), Angus (AN), Red Angus (AR), Gelbvieh (GV), Hereford (HH), Limousin (LM), Shorthorn (SH), and Simmental (SM). Admixed cattle included influenced (breed designation + I) and true first crosses (F1). Influenced cattle were individuals with PBT at least 0.50 but less than 1 and at least 5% more than the next largest (secondary) breed fraction. True F1 groups in the genotyped population included: F1 B × B (F1BB), F1 B × C (F1BC), and F1 C × C (F1CC), where B refers to breeds AN, AR, HH, or SH and C refers to breeds GV or SM. The PBT are colored based on breed origin, where origin 1 (Australia) are dark red, origin 2 (United Kingdom) are green, origin 3 (Continental Europe) are dark yellow, and origin 4 (mixed origins) are black. The 95% confidence ellipses are shaded regions around a given PBT; File S4: Graphical representation of population differentiation estimates (F_ST_’) by genetic marker for purebred primary breed types only (A) or all primary breed types (B). Markers with estimates above 0.25 are dark red. Markers identified in Figure 9B and Figure 10B are yellow.
