# Supplementary figures and images for: Genetic Distinctness and Diversity of American Aberdeen Cattle Compared to Common Beef Breeds in the United States
Source: Genes (Basel). 2023 Sep 22;14(10):1842. doi: 10.3390/genes14101842 (PMC10606367; doi:10.3390/genes14101842)

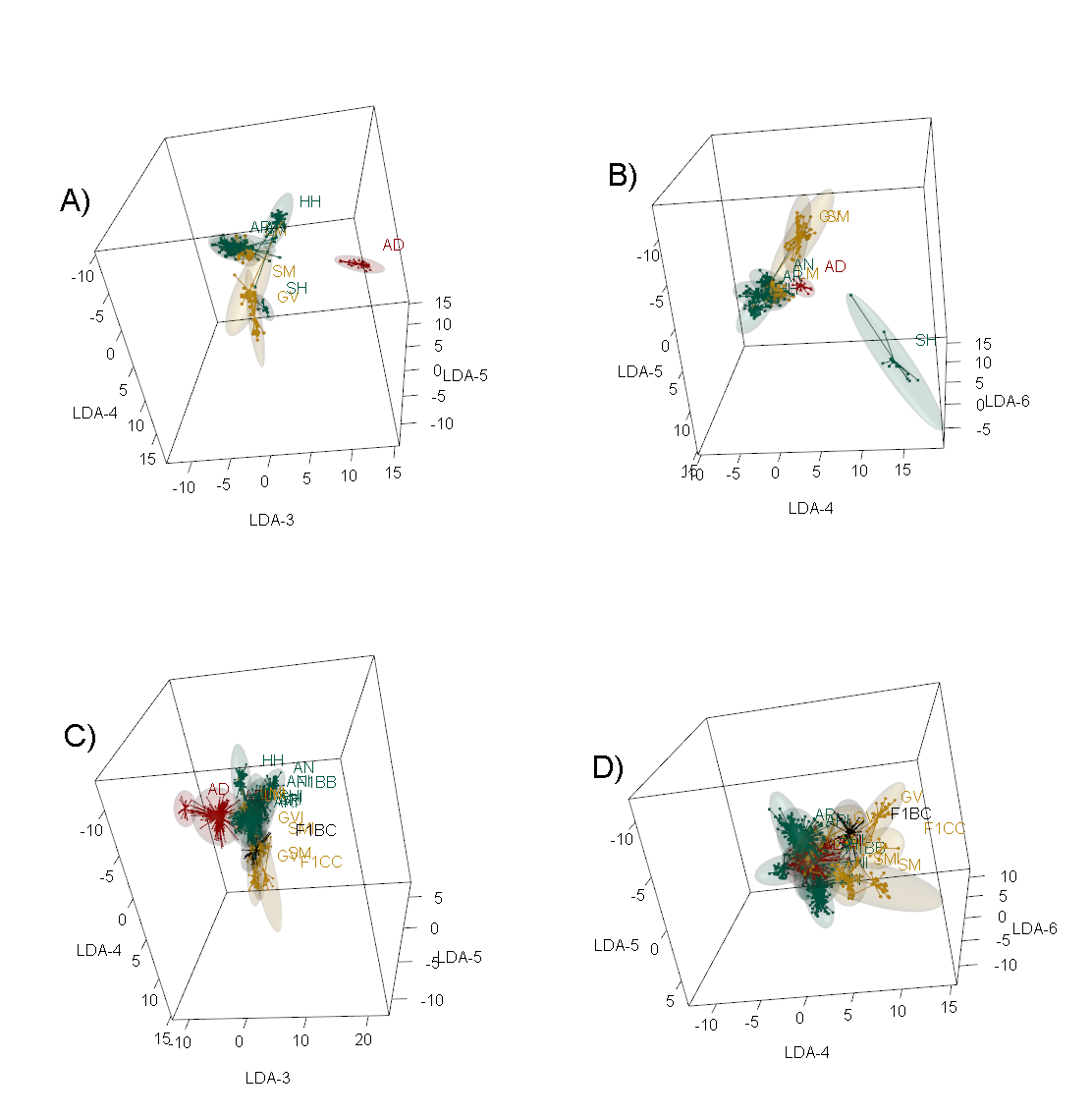

Supplement: Supplementary file 1 [file genes-14-01842-s001.zip › Supplementary File S3.png]

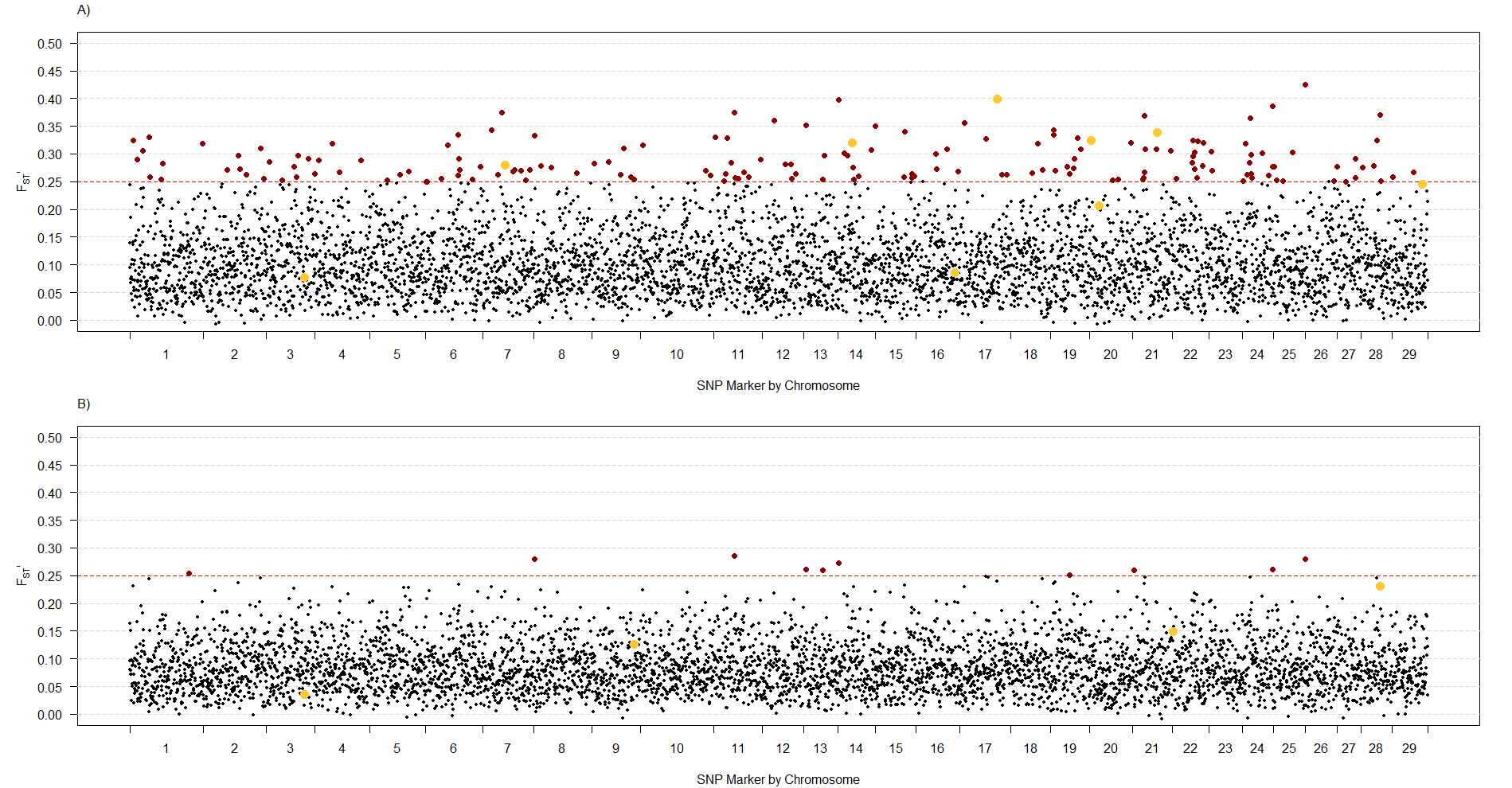

Supplement: Supplementary file 1 [file genes-14-01842-s001.zip › Supplementary File S4.TIFF]
